# Identification of Protein Tyrosine Phosphatase Receptor Zeta as a Novel Complement C1q Synaptic Interacting Partner

**DOI:** 10.1002/alz.089467

**Published:** 2025-01-03

**Authors:** Matthew John Benskey

**Affiliations:** ^1^ Michigan State University, Grand Rapids, MI USA

## Abstract

**Background:**

The pathological correlate most tightly associated with cognitive decline in AD is synapse loss. The presence of pathological tau significantly correlates with synaptotoxicity and cognitive decline in AD, yet it is currently unclear how pathological tau causes synapse loss. Within the brain, complement component C1q coats the outer membrane of weak or damaged synapses, resulting in the phagocytic removal of tagged synapses by microglia. This process is tightly regulated within healthy individuals to prevent aberrant synaptic pruning. For example, synaptic deposition of C1q requires presentation of a C1q‐binding partner in the outer membrane of weak/damaged synapses. In contrast, C1q synaptic deposition is increased in the brains of AD patients where it colocalizes with pathological tau. C1q synaptic deposition is also increased in animal models of tauopathy and blocking C1q synaptic deposition in these models rescues synapse loss. However, it is currently unclear how C1q can recognize tauopathy affected synapses. The goal of the current study is to identify molecules that recruit C1q to AD‐affected synapses.

**Method:**

To identify proteins that facilitate C1q synaptic deposition in response to tauopathy we immunoprecipitated (IP’d) C1q from postmortem human AD (Braak stage V‐VI) or control (Braak stage I‐II) frontal cortex tissue and performed unbiased mass spectrometry to identify proteins that Co‐IP’d with C1q. Identified proteins were compared between AD and controls to identify disease specific C1q interactors, after which AD‐specific C1q interacting proteins were analyzed to identify transmembrane proteins that localize to the synapse. To validate C1q interactors identified by mass spec, IPs were repeated, and elution’s were immunoblotted for the identified interactor.

**Result:**

From this analysis we have identified protein tyrosine phosphatase receptor zeta (PTPRZ) as a novel C1q synaptic interacting protein. Subsequent validation analysis revealed that PTPRZ interacts with oligomeric tau in postmortem human AD tissue and is also increased in the hippocampus and cortex of PS19 mice.

**Conclusion:**

In conclusion, we present data identifying and validating a novel AD‐associated C1q synaptic binding partner. Future studies will determine if preventing the synaptic presentation of PTPRZ will rescue synapse loss in models of tauopathy.

